# Retinol-binding protein type 1 expression predicts poor prognosis in head and neck squamous cell carcinoma

**DOI:** 10.1186/s12885-024-12565-3

**Published:** 2024-10-15

**Authors:** Ling-ling Fu, Ming Yan, Xin Yu, Min Shao, Martin Gosau, Reinhard E. Friedrich, Tobias Vollkommer, Ralf Smeets, Hong-chao Feng, Liya Xu

**Affiliations:** 1 Department of Oral and Maxillofacial Surgery, Guiyang Hospital of Stomatology, Guiyang, 050017 PR China; 2 Department of Oral and Maxillofacial Surgery, University Medical Center Hamburg- Eppendorf, Hamburg, 20246 Germany; 3 Department of Oral and Maxillofacial Surgery, Division of Regenerative Orofacial Medicine, University Medical Center Hamburg-Eppendorf, Hamburg, 20246 Germany

**Keywords:** RBP1, Bioinformatic analysis, Prognosis biomarker, Head and neck squamous cell carcinoma

## Abstract

**Background:**

Head and neck squamous cell carcinoma (HNSCC) is the sixth most prevalent malignancy worldwide, with high incidence and poor survival rates. RBP1 is highly expressed in several kinds of cancer and plays a potential prognostic factor. However, the relationship between RBP1 and HNSCC were analyzed based on The Cancer Genome Atlas (TCGA) database.

**Materials and methods:**

RBP1 expression and clinical information were obtained from the Cancer Genome Atlas (TCGA) database. Tumor tissue and adjacent normal tissue of 6 HNSCC patients were collected to analyze the RBP1 mRNA expression level by quantitative PCR. Cox regression analysis was used to evaluate the prognostic values of RBP1 and clinical data in HNSCC. A nomogram was also established to predict the impact of RBP1 on prognosis based on Cox multivariate results. The methylation level of RBP1 in HNSC and its prognosis were analyzed in UALACN and MethSurv. Finally, the potential biological functions of RBP1 were investigated using gene set enrichment analysis (GSEA) and single sample GSEA (ssGSEA).

**Results:**

The mRNA expression levels of RBP1 were highly expressed in HNSCC tissue. The Cox analyses demonstrate that highly-expressed RBP1 is an independent prognosis marker(*P* < 0.05). ROC curve analysis showed that performances of RBP1 (area under the ROC curve: 0.887, sensitivity: 84.1%, specificity: 79.9%). The methylation was increased in HNSCC patients compared with normal subjects(*P* < 0.05) and was associated with better prognosis at sites cg06208339, cg12298268, cg12497564, cg15288618, cg20532370, cg23448348. Additionally, RBP1 expression is mildly associated with immune cell infiltration and immunological checkpoints.

**Conclusion:**

RBP1 is overexpressed and associated with poor patient prognosis in head and neck squamous cell carcinoma.

**Supplementary Information:**

The online version contains supplementary material available at 10.1186/s12885-024-12565-3.

## Introduction

Head and neck tumor is the sixth most common malignancy in the world, with 830 thousand new cases diagnosed annually. Head and neck squamous cell carcinoma (HNSCC) comprises 90% of head and neck tumors [1, 2]. For the past few years, advances in treatment, such as surgery, radiotherapy, and chemotherapy, have contributed to certain improvement in clinical outcomes [3]. However, the 5-year survival rate of HNSCC patients remains 40–50% due to tumor metastasis, recurrence, and drug resistance [4]. Therefore, searching for specific molecular markers of HNSCC and strengthing research into the molecular mechanism underlying its initiation and development are of paramount significance to further improve the early diagnosis and treatment of HNSCC [5–7].

Retinol-binding protein type 1 (RBP1) is a lipocalin protein family member that transports retinol from the liver to epithelial cells and provides the retina with retinol by specifically binding with the retinal epithelial cells [8, 9]. Retinol is also known as vitamin A with implications for the proliferation and differentiation of epithelial cells [10]. RBP1 controls conversion of retinol to retinyl esters and reduces the activity of retinyl esters, thereby affecting retinoic acid metabolism. During embryonic development, retinoic acid can inhibit carcinogenesis and regulate the growth and apoptosis of normal and aberrantly differentiated cells, demonstrating the potential involvement of retinoic acid in the initiation and development of cancer. The up-regulation of RBP1 could promote the proliferation of lung adenocarcinoma cell line A549, and contribute to epithelial-mesenchymal transition by up-regulate CK5, CK6, CK14, CK17, RAR-α and down regulate RARβ [11]. It was reported that downregulated RBP1 was associated with the occurrence of prostate cancer, endometrial cancer, and ovarian cancer, etc., while upregulated RBP1 was correlated with the occurrence of lung adenocarcinoma and laryngeal cancer, etc [12–15].

Wu et al. experimented with RBP1-deficient mice and found an increased death rate than normal mice, a trend toward reduced blood count and platelet, and development of myelofibrosis and spleen and liver enlargement in partial mice, [16]. Gao et al. proved that RBP1 overexpression was relevant to the malignant phenotype of oral squamous cell carcinoma, which was attributed to the deactivation of the RBP1-CKAP4 axis-mediated autophagy [9]. However, there are no relevant studies concerning the relation between RBP1 and HNSCC.

In this study, the expression of RBP1 in HNSCC was analyzed based on the RNA-seq data from TCGA database, and it was verified in clinical tissue samples with the PCR method. The correlation between RBP1 expression and the prognosis of HNSCC patients was analyzed via Kaplan-Meier survival analysis, univariate and multivariate analyses. In addition, a nomogram was plotted to discuss the potential diagnostic and prognostic values of RBP1. Gene set enrichment analysis (GSEA) was applied to explore the potential biological function of RBP1. Overall, this study identified that RBP1 is an independent prognostic factor of HNSCC.

## Methods

### Data acquisition

All original data were acquired from The Cancer Genome Atlas (TCGA) (https://cancergenome.nih.gov/) and Gene Expression Omnibus (GEO) (https://www.ncbi.nlm.nih.gov/geo/) databases. Level-3 HTSeq-FPKM data of HNSCC patients, including 44 normal and 502 tumor cases, were downloaded from TCGA data portal (http://tcga-data.nci.nih.gov/tcga/), then were transformed into transcripts per million reads (TPM) using the equation TPM= [FPKM(i)/sum (FPKM all transcripts)] × 10^6^ and log2-transformed for subsequent analyses [17]. Gene expression data were divided into high and low groups according to the median expression levels of RBP1. Both TCGA and GEO were a public open database, the relevant information obtained from there did not require additional ethics approval.

### Quantitative real-time PCR analysis

Oral squamous cell carcinoma tissues and adjacent control tissues were collected from patients who underwent surgical procedures at Guiyang Stomatology Hospital from 2021 to 2022. Total RNA was extracted from using TRIzol Universal Reagent (TIANGEN, Beijing, China) according to the manufacturer’s instructions. RNA concentrations were determined on a Nanodrop2000 Spectrophotometer (Thermo Fischer Scientific, Waltham, MA, USA). Total RNA (2.5 µg) was subjected to cDNA synthesis using a qScript cDNA SuperMix (Quanta Biosciences, Beverly, MA, USA) through the following consequent cycles: firstly at 25 ℃ for 5 min, followed by 42 ℃ for 30 min and finally at 85 ℃ for 5 min. A real-time PCR was performed to determine the mRNA levels of RBP1 and GAPDH using SYBR Green Master MIX (ABI, Vernon, CA, USA). Real-time PCR results were calculated using the 2^−∆∆cq^ method [18].

### Enrichment analysis

Expression profiles (HTSeq-Counts) were compared between the high RBP1 expression group and the low RBP1 expression group to identify DEG using Wilcoxon rank-sum test [19] in the R language-related software, DESeq2 (version 1.26.0). Differences with a |log2 fold change|>1 and adjusted P-value < 0.05 were considered threshold values for identifying DEGs [20]. Gene ontology (GO) enrichment and Kyoto encyclopedia of genes and genomes (KEGG) pathway analyses of the 280 DEGs were performed by “ClusterProfiler” package [21] and visualized by the “ggplot2” package. In addition, the protein-protein interaction network of RBP1 co-expressed genes was visualized by STRING (http://string-db.org; version 11.5) with a minimum level of confidence > 0.4 to analyze the functional interactions among proteins [22].

### Immune infiltration analysis by single-sample GSEA (ssGSEA)

Immune infiltration analysis of HNSCC samples was performed by the ssGSEA method using the GSVA package in R (http://www.biocondutor.org/package/release/bioc/html/GSVA.html) for 24 types of immune cells [23], including neutrophils, mast cells, eosinophils, macrophages, natural killer (NK) cells, CD56dim NK cells, CD56bright NK cells, dendritic cells (DCs), immature DCs (iDCs), activated DCs (aDCs), plasmacytoid DCs (pDCs), T cells, CD8^+^ T cells, T helper (Th) cells, Th1 cells, Th2 cells, Th17 cells, T follicular helper cells, regulatory T cells (Treg), central memory T cells (Tcm), effector memory T cells (Tem), gamma delta T cells (Tgd), cytotoxic cells, and B cells. The Estimation of tumor microenvironments using expression data (ESTIMATE, version 1.0.13), including estimatescore, immuneScore, stromalscore [24]. Spearman correlation was used to calculate the correlation amoung RBP1, immune checkpoints and mismatch [25]. Analyses were performed with R (3.6.0), and visualized with ggplot2 (3.3.3).

### Statistical analysis

GraphPad Prism (GraphPad 9.0 Software) and R software (3.6.3) were used for data analysis. Wilcoxon rank-sum test and Wilcoxon rank signed test was used to analyze the expression of RBP1 in unpaired and paired samples, respectively [26]. In addition, ROC analysis and the frequently-used method for binary assessment were conducted using the pROC package (1.17.0.1) [27] to assess the diagnostic capability of RPB1 in head and neck cancer. The computed AUC value from 0.5 to 1 indicates the discriminative potential from 50–100% [28]. Kaplan–Meier survival curve was used for survival analysis [29]. Univariate and multivariate analyses were carried out based on Cox proportional hazard regression [30]. P-value < 0.05 was considered statistically significant.

## Results

### Relative expression level of RBP1 in HNSCC

**Fig. 1:**
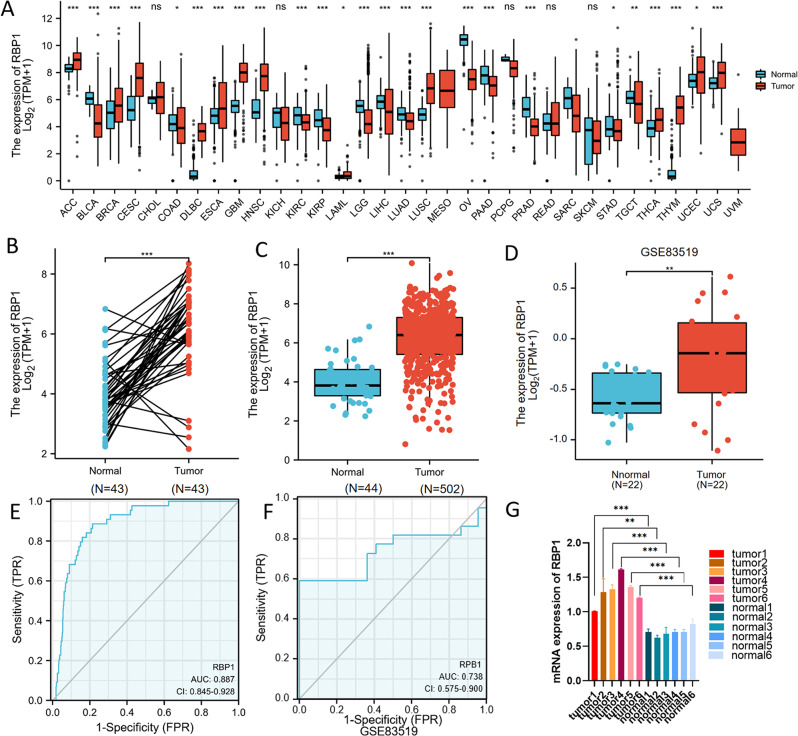
(**A**) RBP1 expression significantly up-regulated in multiple cancers including HNSCC. ACC, Adrenocortical carcinoma; BLCA, Bladder Urothelial Carcinoma; BRCA, Breast invasive carcinoma; CESC, Cervical squamous cell carcinoma and endocervical adeno carcinoma; CHOL, Cholangio carcinoma; COAD, Colon adenocarcinoma; DLBC, Lymphoid Neoplasm Diffuse Large B-cell Lymphoma; ESCA, Esophageal carcinoma; GBM, Glioblastoma multiforme; HNSC, Head and Neck squamous cell carcinoma; KICH, Kidney Chromophobe; KIRC, Kidney renal clear cell carcinoma; KIRP, Kidney renal papillary cell carcinoma; LAML, Acute Myeloid Leukemia; LGG, Brain Lower Grade Glioma; LIHC, Liver hepatocellular carcinoma; LUAD, Lung adenocarcinoma; LUSC, Lung squamous cell carcinoma; MESO, Mesothelioma; OV, Ovarian serous cystadenocarcinoma; PAAD, Pancreatic adenocarcinoma; PCPG, Pheochromocytoma and Paraganglioma; PRAD, Prostate adenocarcinoma; READ, Rectum adenocarcinoma; SARC, Sarcoma; SKCM, Skin Cutaneous Melanoma; STAD, Stomach adenocarcinoma; TGCT, Testicular Germ Cell Tumors; THCA, Thyroid carcinoma; THYM, Thymoma; UCEC, Uterine Corpus Endometrial Carcinoma; UCS, Uterine Carcinosarcoma; UVM, Uveal Melanoma. (**B**) RBP1 expression levels in HNSCC and matched normal tissues. (**C**) RBP1 expression levels in HNSCC and normal tissues. (**D**) RBP1 expression levels were significantly higher in the HNSCC tissues compared to the adjacent peritumoral tissues in GSE83519. (**E**)ROC analysis of RBP1 shows promising discrimination power between tumor and normal tissues based on TCGA. (**F**)ROC analysis of RBP1 shows promising discrimination power between tumor and normal tissues based on GSE83519. (**G**) the level of RBP1 mRNA expression in 6 different head and neck squamous cell carcinoma and normal normal tissues

RBP1 mRNA expression level was analyzed in 33 cancers based on TCGA database. RBP1 was significantly up-regulated in 12 cancers, including head and neck squamous cell carcinoma, esophageal and oropharyngeal (Fig. 1A-C). Furthermore, the RBP1 mRNA expression of HNSCC was also higher than normal tissues both from the GSE85319 and the results of qPCR (Fig. 1D, G). Unfortunately, GSE85319 is missing clinical information to perform validation regarding RBP1 in clinical parameters. In addition, RBP1 expression showed promising discriminative power to identify tumors from normal tissue, with AUC value of 0.887 for TCGA and 0.738 for validation group (GSE83519) (Fig. 1E, F).

### Association of RBP1 mRNA with clinical characteristics

**Fig. 2:**
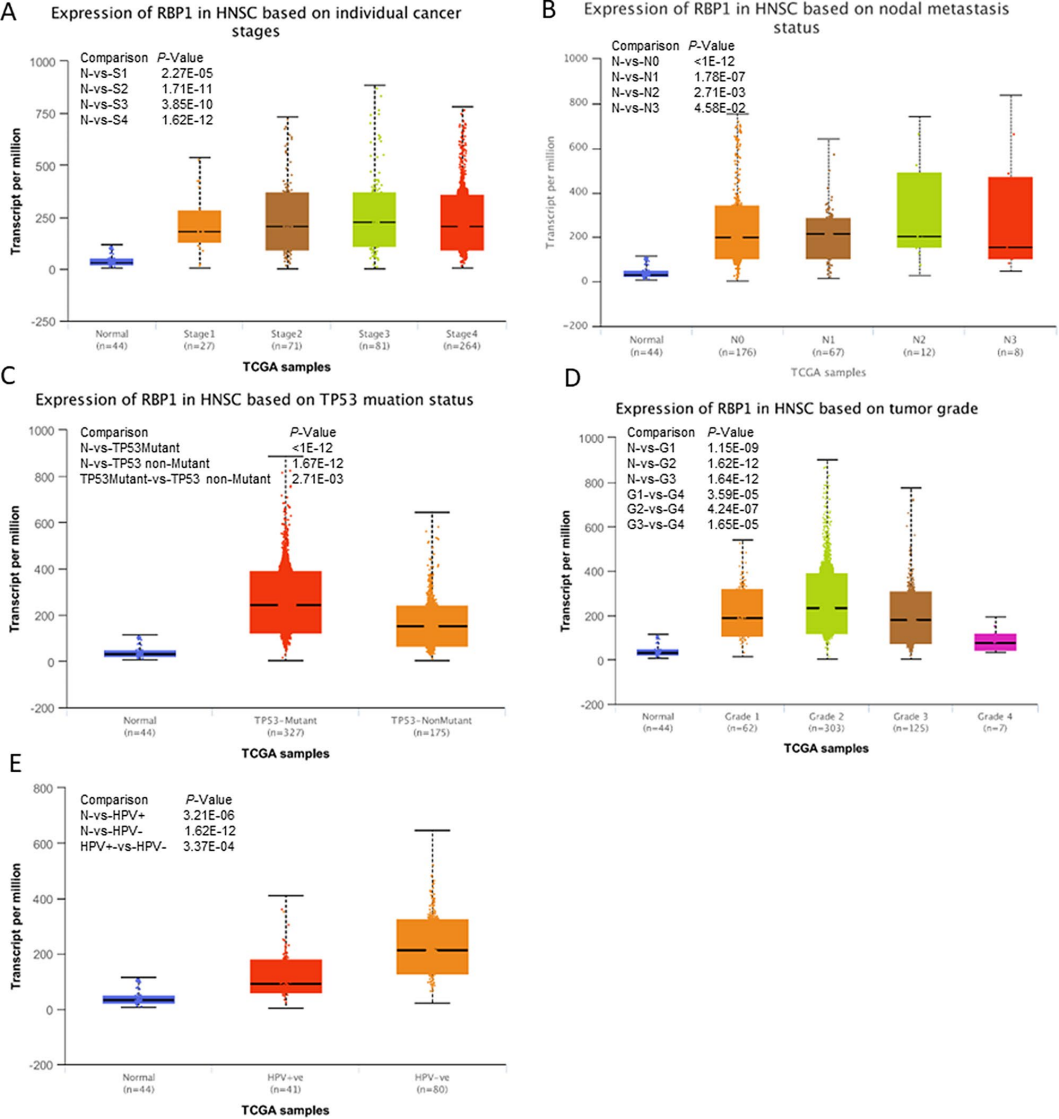
Association of RBP1 expression and clinical characteristics of HNSCC. (**A**) Expression of RBP1 in HNSCC based on individual cancer stages. (**B**) Expression of RBP1 in HNSCC based on nodal metastasis status. (**C**)Expression of RBP1 in HNSC based on TP53 mutation status. (**D**) Expression of RBP1 in HNSCC based on tumor grade. (E) Expression of RBP1 in HNSC based on HPV infection status

The relationship between RBP1 mRNA expression and clinical parameters in HNSCC was assessed by UALCAN [31], which is a comprehensive and interactive web resource for analyzing TCGA transcriptome and clinical patient data by TPM format. The results showed that RBP1 were differentially expressed in patients of different cancer stages, nodal metastasis status, TP53 mutation status, tumor grade, HPV infection status (Fig. 2). In addition, the high RBP1 expression was associated with T stage, histologic grade, age(*P* < 0.05) (Table 1).

**Table 1:** RBP1 expression in HNSCC patients with different clinical parameters

Characteristic	RBP1 mRNA expression		*p*
	Low(*n* = 251)	High(*n* = 251)	
**T stage**			0.031*
T1	17 (3.5%)	16 (3.3%)	
T2	80 (16.4%)	64 (13.1%)	
T3	52 (10.7%)	79 (16.2%)	
T4	98 (20.1%)	81 (16.6%)	
**N stage**			0.339
N0	129 (26.9%)	110 (22.9%)	
N1	34 (7.1%)	46 (9.6%)	
N2	76 (15.8%)	78 (16.2%)	
N3	4 (0.8%)	3 (0.6%)	
**M stage**			0.679
M0	242 (50.7%)	230 (48.2%)	
M1	2 (0.4%)	3 (0.6%)	
**Clinical stage**			0.238
Stage I	9 (1.8%)	10 (2%)	
Stage II	52 (10.7%)	43 (8.8%)	
Stage III	43 (8.8%)	59 (12.1%)	
Stage IV	144 (29.5%)	128 (26.2%)	
**Radiation therapy**			0.686
No	79 (17.9%)	75 (17%)	
Yes	140 (31.7%)	147 (33.3%)	
**Histologic grade**			0.032*
G1	36 (7.5%)	26 (5.4%)	
G2	134 (27.7%)	166 (34.4%)	
G3	66 (13.7%)	53 (11%)	
G4	2 (0.4%)	0 (0%)	
**Lymphovascular invasion**			0.425
No	110 (32.3%)	109 (32%)	
Yes	55 (16.1%)	67 (19.6%)	
**Lymphnode neck dissection**			0.267
No	50 (10%)	40 (8%)	
Yes	198 (39.7%)	211 (42.3%)	
**Gender**			0.762
Female	65 (12.9%)	69 (13.7%)	
Male	186 (37.1%)	182 (36.3%)	
**Age**			0.036*
<=60	110 (22%)	135 (26.9%)	
> 60	140 (27.9%)	116 (23.2%)	

### Prognostic value of RBP1 in HNSCC

**Fig. 3:**
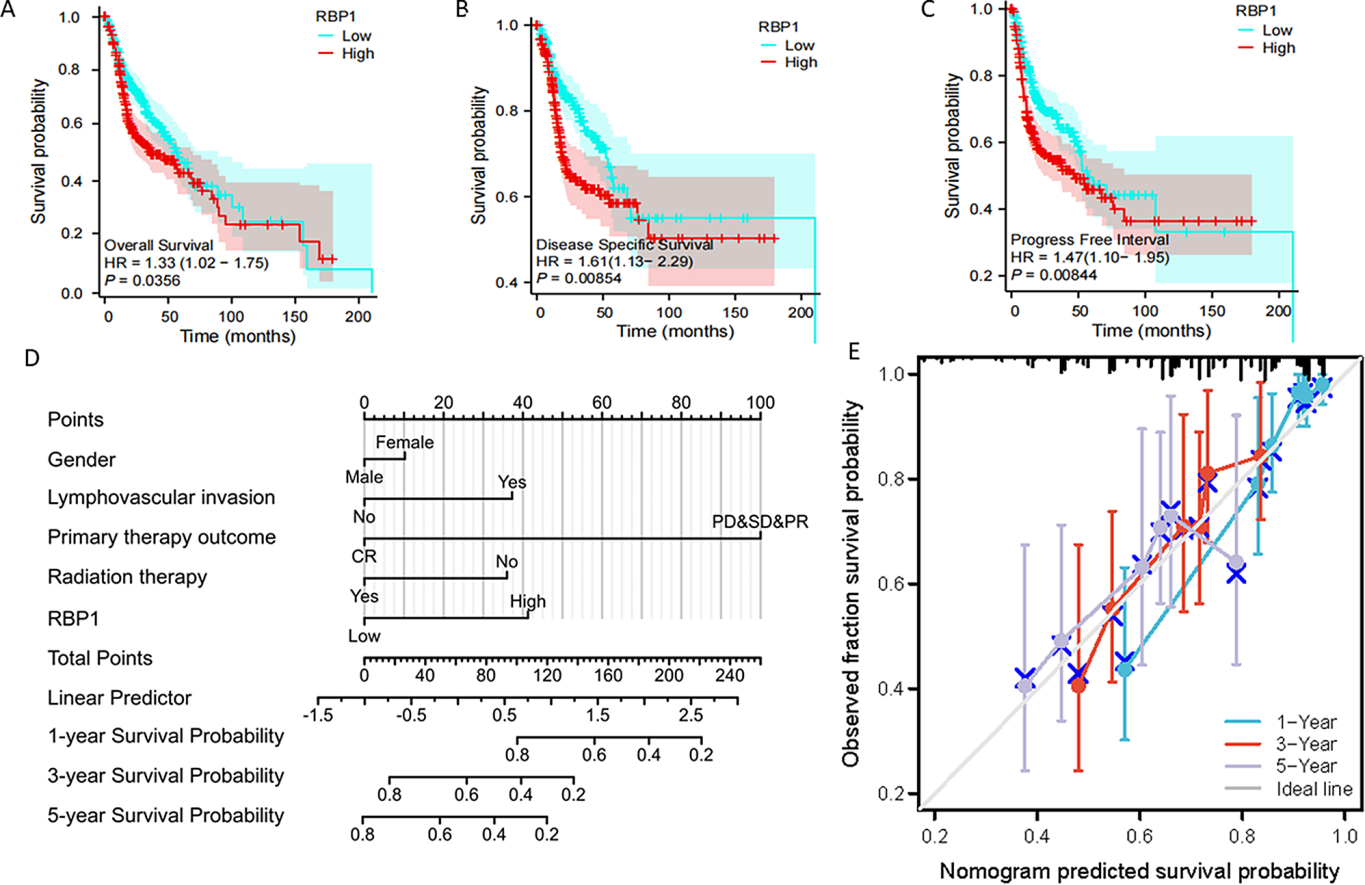
The association of RBP1 expression and prognosis in HNSCC. Kaplan-Meier plotter database analysis shows the differences in (**A**) Progress free interval; (**B**) Disease specific survival; (**C**) Overall survival; (**D**) nomogram integrates RBP1 and other prognostic factors in HNSCC from TCGA data; (**E**) The calibration plot of the nomogram

KM survival curves demonstrated that patients having higher-level RBP1 expression tend to have a poor prognosis performance in terms of progression-free survival (PFI), and disease-free survival (DSS), overall survival (OS) (Fig. 3A–C). To confirm the correlation of RBP1 expression with overall survival (OS), disease-specific survival (DSS) patients, the prognostic factors influencing were identified by Cox regression analysis. Multivariate analyses revealed that RBP1 was an independent prognostic factor for OS in patients with HNSCC (HR = 1.962, 95% CI: 1.277–3.013, *P* = 0.002). Furthermore, primary therapy outcome (HR = 0.202, 95% CI: 0.123–0.332, *P* < 0.001), lymphovascular invasion (HR = 1.764, 95% CI: 1.111–2.801, *P* = 0.016), radiation therapy (HR = 0.529, 95% CI: 0.332–0.841, *P* = 0.007), were also an independent prognostic factor for OS in patients (Table 2). Disease-specific survival (DSS) could better reflect the clinical benefit of disease-specific. Based on the multivariate analysis of prognostic factors, RBP1 was also an independent prognostic factor of DSS (HR = 1.905, 95% CI: 1.143–3.174, *P* = 0.013) (Table 3). Moreover, within the nomogram, RBP1 expression was found to contribute a high data points (ranging from 0 to 100) compared with the other clinical variables, which was consistent with the results of multivariate Cox regression. The C-index of the nomogram was 0.755 with 1000 bootstrap replicates (95% confidence interval: 0.731–0.779). The bias-corrected line in the calibration plot was close to the ideal curve (i.e., the 45-degree line) [32], indicating good agreement between the predicted and observed values (Fig. 3D, E).

**Table 2:** Association of clinicopathological characteristics with overall survival using univariate or multivariate Cox regression analysis

Characteristics	Total	Univariate analysis		Multivariate analysis	
	(*N*)	Hazard ratio (95% CI)	*P* value	Hazard ratio (95% CI)	*P* value
T stage(T1&T2 vs. T4&T3)	486	1.245 (0.932–1.661)	0.137		
N stage(N0 vs. N1&N2&N3)	479	1.263 (0.964–1.653)	0.09	1.421 (0.902–2.240)	0.13
M stage(M0 vs. M1)	476	4.745 (1.748–12.883)	**0.002**	4.726 (0.610-36.602)	0.137
Histologic grade(G1&G2 vs. G3&G4)	482	0.939 (0.688–1.282)	0.692		
Clinical stage(Stage I&Stage II&Stage vs. Stage IV)	487	1.163 (0.886–1.527)	0.277		
Primary therapy(PD&SD&PR vs. CR)	417	0.182 (0.124–0.268)	**< 0.001**	0.202 (0.123–0.332)	**< 0.001**
Radiation therapy(No vs. Yes)	440	0.613 (0.452–0.831)	**0.002**	0.529 (0.332–0.841)	**0.007**
Age( < = 60 vs. >60)	501	1.252 (0.956–1.639)	0.102		
RBP1(Low vs. High)	501	1.335 (1.020–1.747)	**0.036**	1.962 (1.277–3.013)	**0.002**
Lymphovascular invasion(No vs. Yes)	340	1.699 (1.211–2.384)	**0.002**	1.764 (1.111–2.801)	**0.016**
Lymphnode neck dissection(No vs. Yes)	498	0.731 (0.526–1.016)	0.062	0.617 (0.273–1.394)	0.246

**Table 3:** Association of clinicopathological characteristics with disease-specific survival using univariate or multivariate Cox regression analysis

Characteristics	Total	Univariate analysis		Multivariate analysis	
	(*N*)	Hazard ratio (95% CI)	*P* value	Hazard ratio (95% CI)	*P* value
T stage(T1&T2 vs. T4&T3)	461	1.459 (0.988-2.153)	0.057	2.150 (1.113-4.151)	**0.023**
N stage(N0 vs. N1&N2&N3)	454	1.485 (1.044-2.112)	0.028	1.053 (0.613-1.809)	8.056
M stage(M0 vs. M1)	447	8.056 (2.527-25.680)	<0.001	0.121	8.056
Histologic grade(G1&G2 vs. G3&G4)	462	1.051 (0.712-1.552)	0.801		
Clinical stage(Stage I&Stage II&Stage vs. Stage IV)	462	1.170 (0.822-1.666)	0.383		
Primary therapy(PD&SD&PR vs. CR)	405	0.094 (0.061-0.146)	<0.001	0.094 (0.055-0.163)	**<0.001**
Radiation therapy(No vs. Yes)	424	0.740 (0.492-1.112))	0.147		
Age(<=60 vs. >60)	476	1.078 (0.763-1.524)	0.670		
RBP1(Low vs. High)	476	1.492 (1.051-2.118)	0.025	1.905 (1.143-3.174)	**0.013**
Lymphovascular invasion(No vs. Yes)	326	1.658 (1.079-2.546)	0.021	1.146 (0.678-1.939)	0.610
Lymphnode neck dissection(No vs. Yes)	473	0.719 (0.465-1.114)	0.14		

### Gene set enrichment analysis

**Fig. 4:**
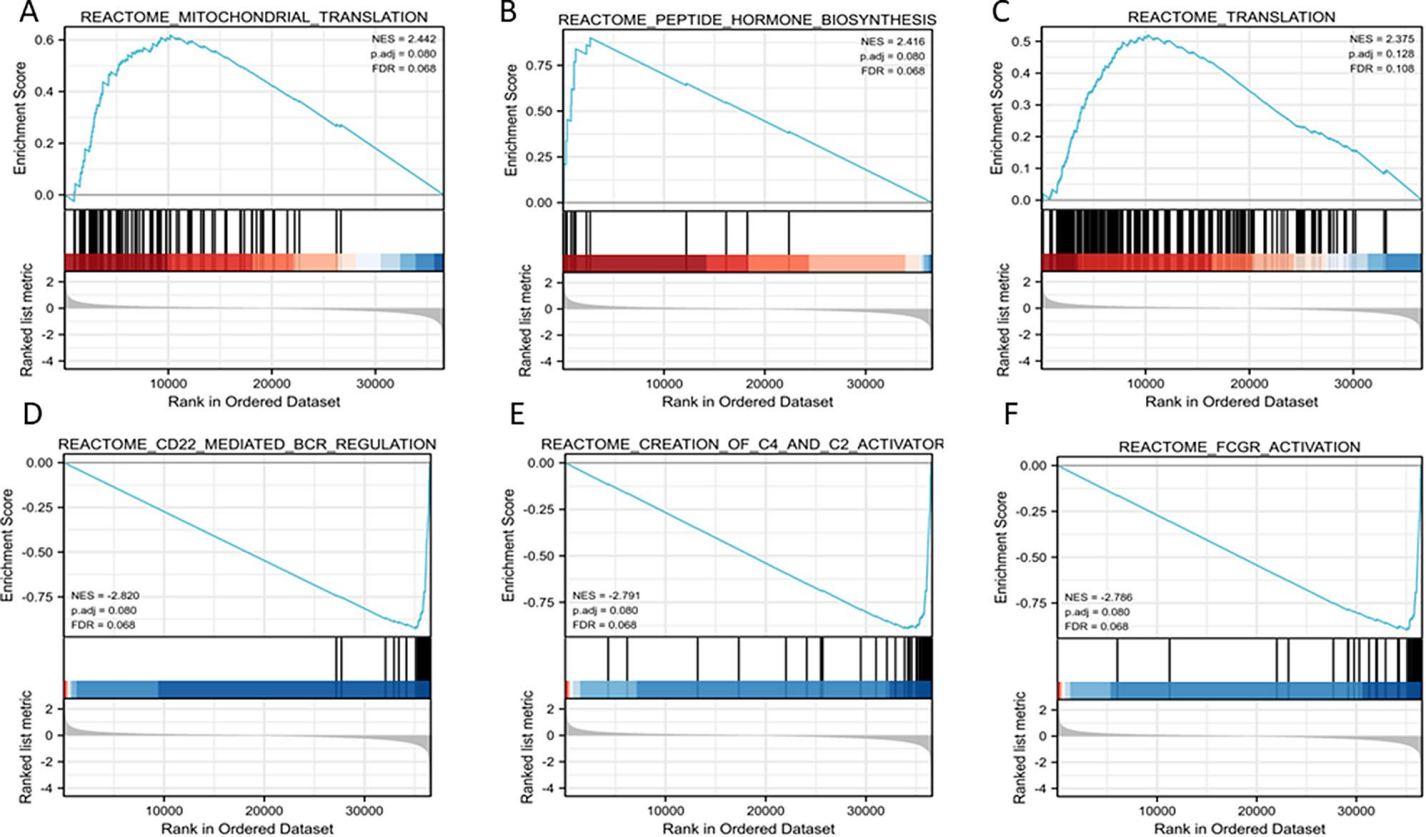
Ecrichment plots of RBP1 in HNSCC from GSEA. (**A**) Mitochondrial translation; (**B**) Peptide hormone biosynthesis; (**C**)Translation; (**D**) CD22 mediated BCR regulation; (**E**) Creation of C4 and C2 activator; (**F**) FCGR activation

Based on significant differences (P-value < 0.05, FDR < 0.25), GSEA was used to identify signaling pathways associated with HNSCC between the high and low RBP1 expression groups, including 265 positive regulation pathways and 212 negative regulation pathways. The most significantly enriched pathways were the mitochondrial translation, peptide hormone biosynthesis, translation, CD22 mediated BCR regulation, creation of C4 and C2 activators, FCGR activation in positive and negative respectively (Fig. 4).

### Correlation between RBP1 and immune infiltration, immune checkpoints, mismatch repair genes in HNSCC

**Fig. 5:**
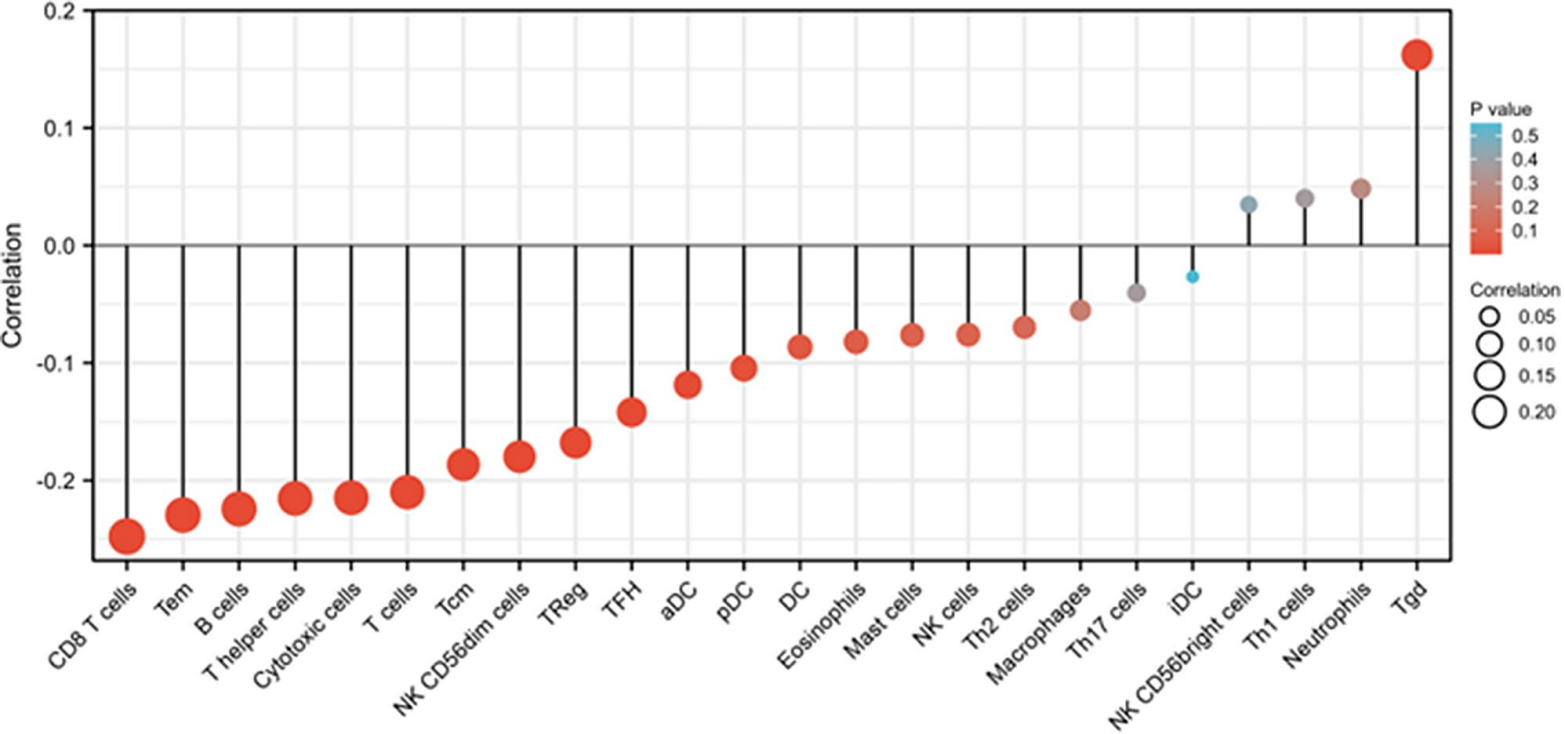
Correlations between the relative abundance of 24 immune cells and RBP1 expression levels. The size of the dots represents the absolute Spearman’s correlation coefficient values

**Fig. 6:**
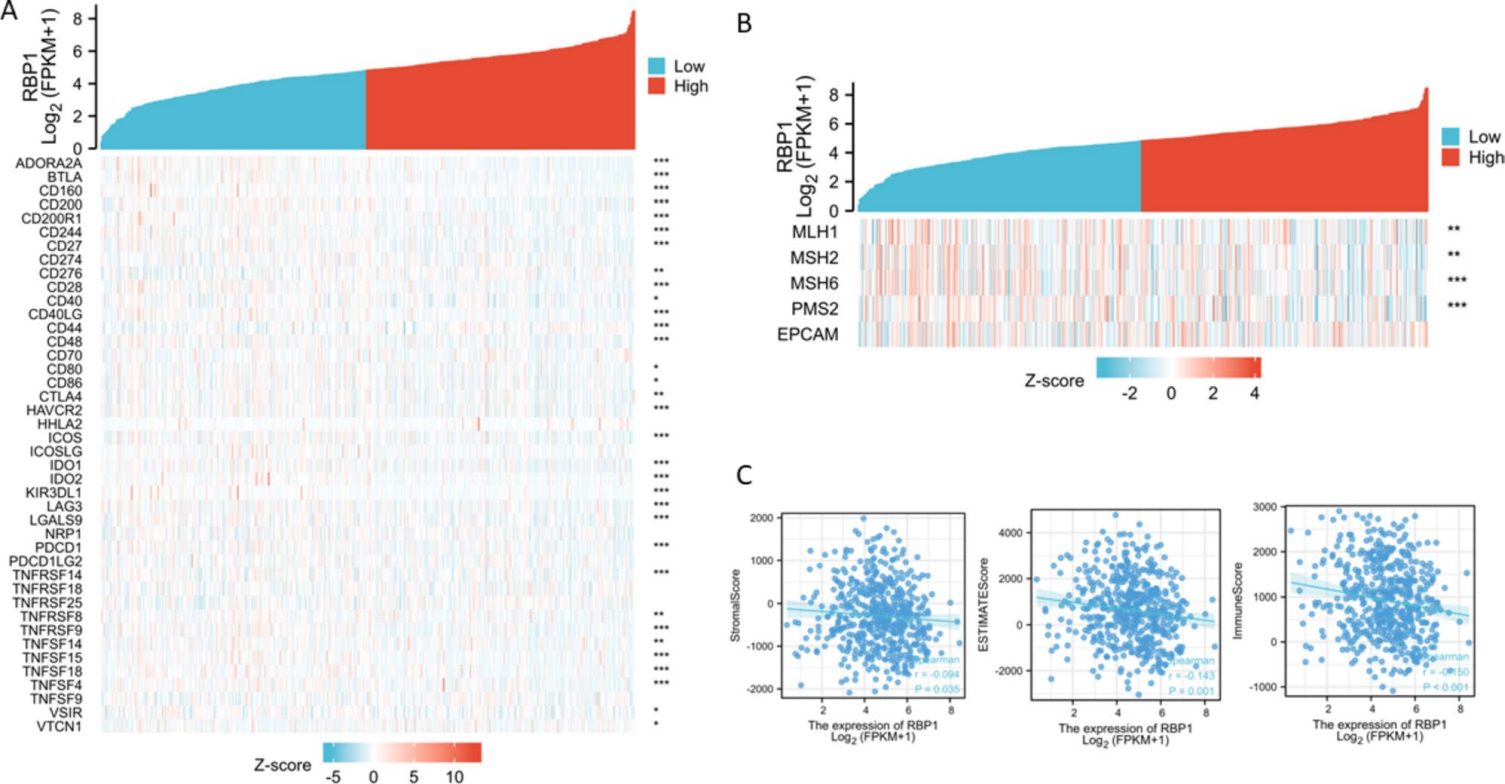
Associations between RBP1 expression and tumor immune infiltration. (**A**) Associations between RBP1 expression and immune checkpoint molecules in HNSCC from TCGA dataset; (**B**) Associations between RBP1 expression and mismatch repair genes in HNSCC from TCGA dataset; (**C**) Associations between RBP1 expression and tumor microenvironment in HNSCC from TCGA dataset; *P < 0.05; **P < 0.05; ***P < 0.001

The association between RBP1 expression and immune cell infiltration was analyzed using single-sample Gene Set Enrichment Analysis (ssGSEA). RBP1 expression was negatively correlated with the abundance of aDC, B cells, CD8^+^ T cells, Cytotoxic cells, NK CD56dim cells, pDC, T cells, T helper cells, Tcm, Tem, TFH, TReg, and was positively correlated with the abundance of Tgd. (Fig. 5). Pearson’s method was used to estimate the correlations between the RBP1 expression and immune checkpoint molecules, mismatch repair genes, tumor microenvironment. RBP1 was related to ImmuneScore, ESTIMATEScore, StromalScore (*P* < 0.05, Fig. 6C). Co-expression analysis of RBP1 and immune Checkpoint molecules indicated that RBP1 was significantly associated with PDCD1, CTLA4 etc. (*P* < 0.05, Fig. 6A). Co-expression analysis of RBP1 and mismatch repair genes indicated that RBP1 was related to MLH1, MSH2, MSH6, PMS2 in HNSCC (*P* < 0.05, Fig. 6B).

### Functional annotation and predicted signaling pathways

**Fig. 7:**
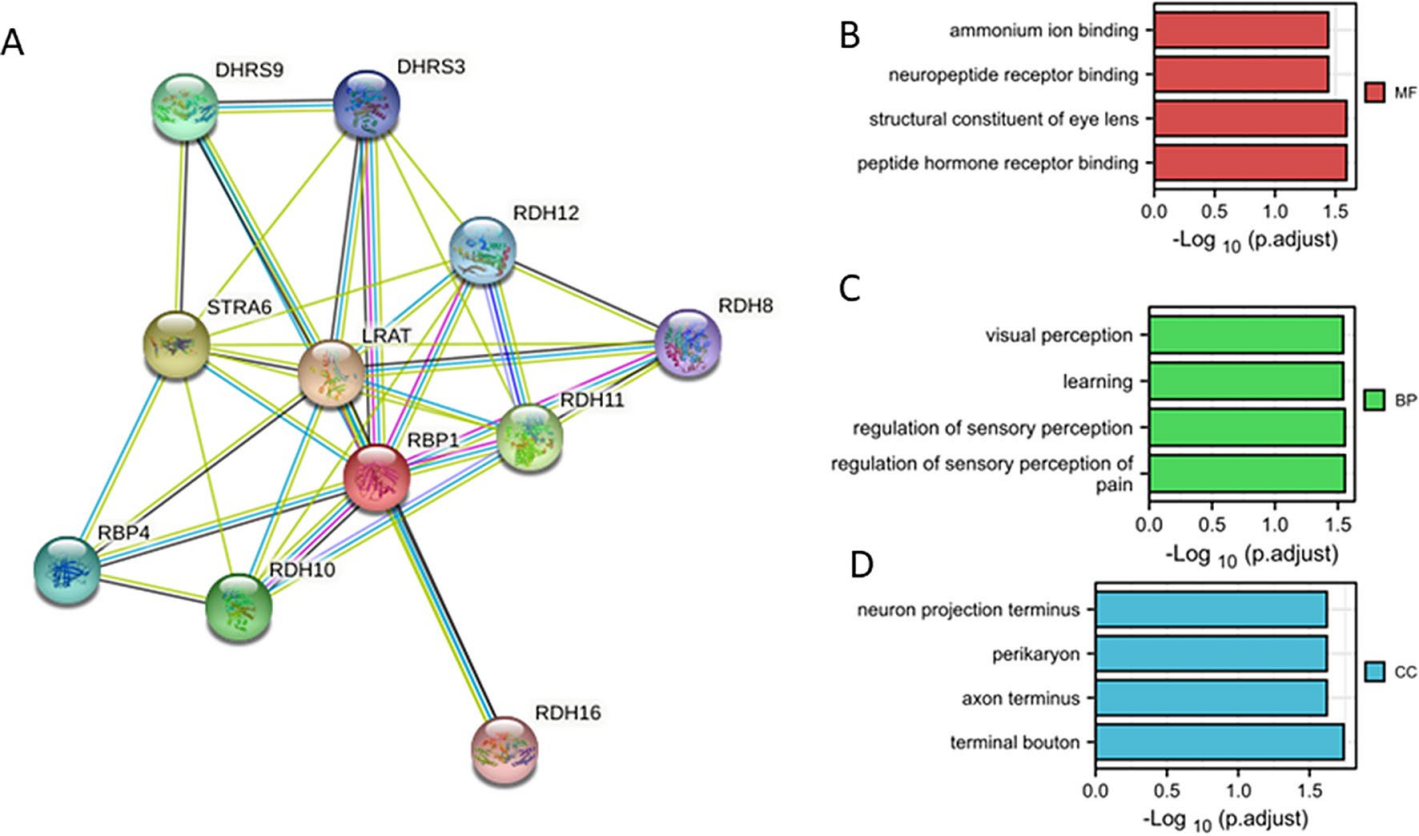
Protein–protein interaction network and GO enrichment in HNSCC. (**A**) RBP1 interaction protein plot and description. (**B**) The top 5 GO enrichment terms in MF. (**C**) The top 5 GO enrichment terms in BP.(**D**) The top 5 GO enrichment terms in CC

The top ten co-expression genes of RBP1 were shown in Fig. 7A, and the most related gene was LRAT [33], is considered to have a cancer-promoting effect. To better understand the functional implication of RBP1 in HNSCC from the 280 DEGs identified between the high and low expression groups, GO enrichment analysis was performed using the ClusterProfile package. Furthermore, 12 enriched terms were identified in the GO “biological process” category, the top four items were ammonium binding, neuropeptide receptor binding structural constituent of eye lens, peptide hormone receptor, binding (Fig. 7B); 14 enriched terms were identified in the GO “biological process” category, including visual perception, learning, regulation of sensory perception regulation of sensory perception of pain (Fig. 7C). 12 enriched terms within the “cellular component” category were associated with the activation of multiple proteins (Fig. 7D). These were analyzed for correlation of RBP1 and TP53, CDKN2A, CCND1, PTEN in TCGA database, that revealed RBP1 was positively correlated with CCND1 and negatively correlated with TP53, CDKN2A (Supplement Fig. 1).

### Bioinformatical analysis

**Fig. 8:**
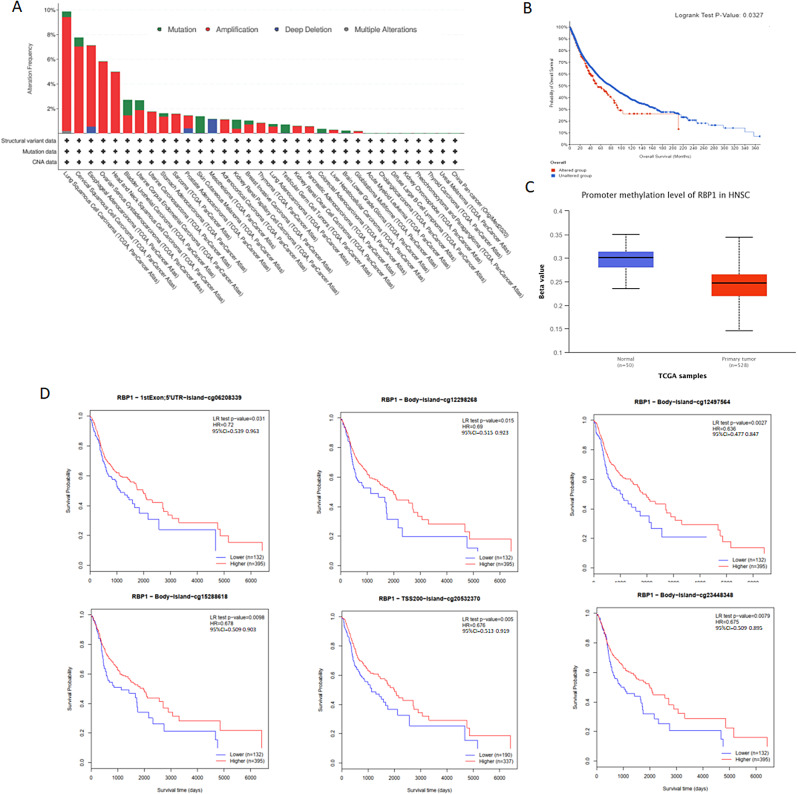
Mutation feature of RBP1 in HNSCC from TCGA cohort using the cBioPortal tool; (**A**) The alteration frequency with mutation type of RBP1 in different tumor samples from TCGA cohorts; (**B**) K-M survival analysis of OS with or without RBP1 alteration; (**C**) The methylation level of RBP1 in HNSCC; (**D**) The Kaplan-Meier survival of the promoter methylation of RBP1S

The correlation between RBP1 expression and its mutation in pan-cancer was analyzed using cBioPortal, as revealed in Fig. 8A. In 523 HNSCC cases, the genetic alteration was found in 26 cases, and the mutation rate was 4.97%. Besides, RBP1 alteration in HNSCC was associated with a shorter overall survival, implying that the genetic mutation of RBP1 could also affect HNSCC patients’ prognosis (Fig. 8B). The methylation level of RBP1 in HNSCC was examined by UALCAN based on TCGA. The methylation was higher in normal group (Fig. 8C). MethSurv analysis showed that patients with high RBP1 methylation had a better overall survival than patients with low RBP1 methylation. The 6 CpG sites are shown in Fig. 8D, each hazard ratio and 95% confidence interval are shown in supplement Table 1.

## Discussion

Pan-cancer analysis demonstrated that RBP1 mRNA expression was significantly up-regulated in ACC, BRCA, CESC, DLBC, ESCA, GBM, HNSC, LUSC, THCA, THYM, UCEC, and UCS. PCR result showed significantly higher RBP1 mRNA expression in HNSCC tissues than adjacent normal tissues, and correlation analysis revealed a close relationship between high RBP1 expression and advanced cancer stage and tumor grade. TP53 plays a key regulatory role in apoptosis and cell cycle, and mutations in the TP53 gene are considered as the earliest and indispensable event in the initiation and development of HNSCC [34–36]. TCGA data found that RBP1 expression was relevant to the TP53 mutation status in HNSCC patients. TP53, CDKN2A, CCND1, PTEN are considered important driver gene of head and neck squamous cell carcinoma [37, 38]. The above evidence indicates that RBP1 is negatively correlated with tumor suppressors TP53, CDKN2A and positively correlated with cancer-promoting gene CCND1. This type of correlation imply close coordination between RBP1 and other oncogenes in the development of squamous cell carcinoma. Beyond this, the result indicates that high-risk HPV infection is one of the contributing factors for OSCCs. RBP1 had a much higher expression level in HPV negative (−) patients, this means is not typically associated with HPV infection, more experiments are needed to verify. Previous studies revealed that RBP1 affected cell differentiation and tumor progression through interfering with retinoic acid metabolism by decreasing retinol transport, preventing retinyl esters formation, and reducing retinoic acid receptors activity [9, 39–41]. It was also reported that high expression of RBP1 is associated with bladder cancer, tongue cancer and laryngeal squamous-cell carcinoma. Gao et al. proved that RBP1 overexpression was relevant to the malignant phenotype of oral squamous cell carcinoma, which was attributed to the deactivation of the RBP1-CKAP4 axis-mediated autophagy [9]. Taken together, high expression of RBP1 may be associated with the initiation and development of HNSCC.

The present study also identified the remarkable prognostic value of RBP1 for the survival outcomes of HNSCC patients. Kaplan-Meier analysis revealed that higher expression of RBP1 indicated worse OS, PFI, and DSS in HNSCC patients, implying RBP1’s potential as a tumor gene. Moreover, RBP1 was confirmed as an independent prognostic factor for the OS and DSS of HNSCC patients via univariate and multivariate COX regression analyses [42]. In all, RBP1 might be a potential therapeutic target of HNSCC. Nomogram is generated from the multi-variable model that predicts the probability of the occurrence or outcome of a certain disease, which is reliable and effective [43, 44]. It has been extensively applied in clinical prognosis and decision-making. In this study, a nomogram combining RBP1 expression level, primary therapy outcome, lymphovascular invasion, and gender was established, in which RBP1 expression level contributed most, indicating that RBP1 is an effective prognostic factor.

Generally, the the closer the value of the C-index is to 1, the higher the accuracy of the predictive ability of the nomogram. The C-index for OS prediction was 0.755 with higher credibility than our previous study about DCBLD1(C-index was 0.720) [7]. Liang constructed a prediction model for HNSCC, and the C-index was 0.687 in their nomogram [5].

In addition, the present study also explored the role of mutation and promoter methylation of the RBP1 gene in HNSCC prognosis. RBP1 mutations ars associated with poor prognosis in HNSCC. DNA methylation is the covalent modification of cytosine at 5′ site catalysed by DNA methyl transferases, which play an important role in regulating gene activity and transcript levels without changing gene sequence [45]. Studies have reported that DNA methylation is associated with tumor occurrence and development [46, 47]. Chou observed that glioma patients with RBP1 hypermethylation are associated with a better prognosis, and the expression of RBP1 was associated with hypermethylation [48]. We analyzed the methylation level of RBP1 in HNSCC and found that RPB1 promoter methylation was much more prevalent in normal tissue than in HNSCC tissue. Moreover, RBP1 methylation is associated with good prognosis of HNSCC, which was consisted with chou’s study. Esteller reported that transcriptional silencing of RBP1 was associated with CpG island promoter hypermethylation in HN12(head and neck cancer cell lian), Shigeru Tsunoda et al. found that RBP1 showed more frequent methylation in the tumor than the matched proximal resection margin of uninvolved esophagus [12, 49].

Tumor microenvironment is of vital importance in the development and treatment of tumors [50]. Research showed that massive infiltration of immune cells in the tumor tissues was substantially associated with the prognosis of patients [51]. The present study analyzed the relationships between RBP1 and the tumor microenvironment, tumor immune infiltration, immune checkpoint molecules, and mismatch repair genes. Previous studies found that the tumor infiltration of B cells and CD8^+^ T cells was relevant to the poor prognosis of HNSCC patients [52–54]. Moreover, higher expression of RBP1 led to shorter OS in patients, and RBP1 might impact the prognosis of HNSCC patients via affecting the tumor microenvironment.

However, the prediction of single gene still had some limitations and is obviously not as good as multigene. The initiation and progression of tumors result from the mutational synergism of genes with similar functions. In contrast, there was significant tumor suppression in monogenic therapy. For example, p53 was predictive of a cisplatin-based therapeutic benefit in patients with head and neck squamous cell carcinoma [55]. Pembrolizumab as PD-L1 inhibitor have established clinical use. Therefore, single gene analysis still plays a significant role in anticancer treatment [56].

To conclude, this study validated the significantly upregulated expression of RBP1 in HNSCC and identified it as an independent risk factor for the prognosis of HNSCC patients. RBP1, therefore, has potential research value in HNSCC targeted therapy.

## Conclusion

The relationship between RBP1 expression and HNSCC was described for the first time based on TCGA database. Our study found that RBP1 is a potential prognostic factor for survival outcomes. Besides, the RBP1 methylation is correlated with better prognosis of HNSCC. RBP1 is expected to be a novel diagnostic and prognostic factor for HNSCC.

## Electronic supplementary material

Below is the link to the electronic supplementary material.

Supplementary Material 1

Supplementary Material 2

## Data Availability

The datasets analyzed during the current study are publicly available from the following online databases: TCGA ( https://tcga-data.nci.nih.gov/tcga/).

## Abbreviations

GO: Gene ontology
GSEA: Gene set enrichment analysis
FDR: False discover rate
AUC: Area under the curve
C-index: Concordance index
CI: Confidence interval
ROC: Receiver operating characteristic
ssGSEA: Single-sample gene set enrichment analysis
TCGA: The cancer genome atlas
HPA: Human protein atlas
OS: Overall survival
DSS: Disease specific survival
Th1: Type-1 T helper cells
Th2: Type-2 T helper cells
Th17: Type-1 T helper cells
Treg: Regulatory T cell
iDCs: Immature dendritic cells
NK cells: Natural killer cells
Tem: T effector memory
Tcm: T central memory
Tfh: T follicular helper
Tgd: T gamma delta
aDC: Activated DC
HNSCC: Head and neck squamous cell carcinomas
OSCC: Oral squamous cell carcinomas
PD-1: Programmed death protein 1
PD-L1: Programmed death protein ligand 1
