# Supplementary material for: Retinol-binding protein type 1 expression predicts poor prognosis in head and neck squamous cell carcinoma
Source: BMC Cancer. 2024 Oct 15;24:1277. doi: 10.1186/s12885-024-12565-3 (PMC11476480; doi:10.1186/s12885-024-12565-3)
Supplement: Supplementary file 1 — Supplementary Material 1 [file 12885_2024_12565_MOESM1_ESM.docx]

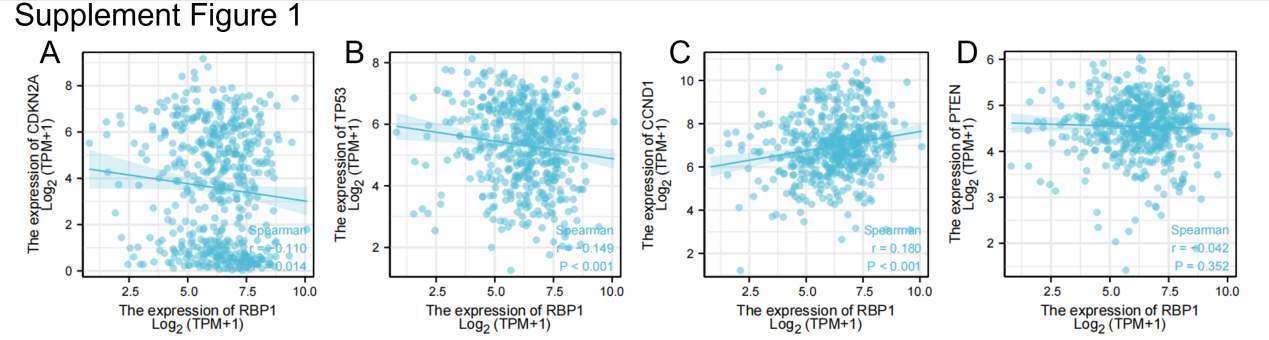


Supplement figure 1. The correlation of mRNA expression between RBP1 and HNSCC driver genes. (A) Associations between RBP1 expression and CDKN2A. (B)Associations between RBP1 expression and TP53. (C)Associations between RBP1 expression and CCND1. (D) Associations between RBP1 expression and PTEN.
