# Supplementary material for: Retinol-binding protein type 1 expression predicts poor prognosis in head and neck squamous cell carcinoma
Source: BMC Cancer. 2024 Oct 15;24:1277. doi: 10.1186/s12885-024-12565-3 (PMC11476480; doi:10.1186/s12885-024-12565-3)
Supplement: Supplementary file 2 — Supplementary Material 2 [file 12885_2024_12565_MOESM2_ESM.docx]

****Supplement table 1. Effect of hypermethylation level on HNSCC prognosis****

| **Hypermethylation id** | **HR** | **95% CI** | **P** |
| --- | --- | --- | --- |
| cg06208339 | 0.720 | (0.539;0.963) | 0.026 |
| cg12298268 | **0.690** | **(0.515;0.923)** | **0.012** |
| cg12497564 | **0.636** | **(0.477;0.847)** | **0.001** |
| cg15288618 | **0.678** | **(0.509;0.903)** | **0.008** |
| cg20532370 | **0.686** | **(0.513;0.919)** | **0.012** |
| cg23448348 | **0.675** | **(0.509;0.895)** | **0.006** |
